# Normal-Weight Obesity and Hidden Cardiometabolic Risk in Young Adults: Implications Beyond BMI-Based Classification

**DOI:** 10.3390/medsci14030354

**Published:** 2026-06-27

**Authors:** Alberto Ramírez Gallegos, Pedro Juan Tárraga López, Mónica Silu Piña Dabreu, Lluis Rodas Cañellas, Ángel Arturo López-González, José Ignacio Ramírez-Manent

**Affiliations:** 1 Primary Care, Balearic Islands Health Service, 07010 Palma, Spain; alberto.ramirez@ibsalut.es (A.R.G.); joseignacio.ramirez@ibsalut.es (J.I.R.-M.); 2 Faculty of Medicine, University of Castilla La Mancha (UCLM), 02008 Albacete, Spain; pjtarraga@sescam.jccm.es; 3 ADEMA-University School, University of the Balearic Islands, 07009 Palma, Spain; m.pina@eua.edu.es (M.S.P.D.); ll.rodas@eua.edu.es (L.R.C.)

**Keywords:** normal-weight obesity, body mass index, cardiometabolic risk, waist-to-height ratio, triglycerides, high-density lipoprotein cholesterol

## Abstract

Background: BMI is widely used for obesity classification, yet it does not adequately reflect adiposity distribution and related metabolic risk. Normal-weight obesity (NWO), defined as excess adiposity despite normal BMI, has emerged as a clinically relevant phenotype associated with increased cardiometabolic risk. However, its prevalence and implications in young populations remain insufficiently characterized. Objective: To evaluate the prevalence of normal-weight obesity and its association with cardiometabolic risk markers in a cohort of young adults undergoing occupational health assessments. Methods: A cross-sectional study was conducted in 12,874 adults aged 22–30 years undergoing routine occupational health assessments. NWO was defined using a strict criterion (normal BMI with a waist-to-height ratio ≥ 0.5) and an expanded definition including additional adiposity markers derived from body fat percentage and visceral fat estimates. Anthropometric, adiposity-related, biochemical, and lifestyle data were collected using standardized protocols. Multivariable logistic regression models adjusted for age and sex were used to assess associations between NWO and cardiometabolic risk markers. Results: Among individuals with normal BMI (*n* = 9290), the prevalence of NWO was 1.79% (*n* = 166) using the strict definition and 2.30% (*n* = 214) with the expanded definition. Compared with metabolically healthy normal-weight individuals, those with NWO exhibited higher triglycerides, fasting glucose, and atherogenic lipid indices, along with lower HDL cholesterol (all *p* < 0.05). In multivariable analyses, NWO was independently associated with elevated triglycerides (OR 4.99; 95% CI 2.90–8.58), an unfavorable triglyceride-to-HDL ratio (OR 7.44; 95% CI 5.19–10.65), and impaired fasting glucose (OR 3.87; 95% CI 2.19–6.82). Associations were consistent across sensitivity and sex-stratified analyses. Conclusions: Normal-weight obesity is present in a measurable proportion of young adults and is associated with an unfavorable cardiometabolic profile despite normal BMI. The triglyceride-to-HDL ratio was consistently associated with normal-weight obesity and showed marked differences across adiposity-defined phenotypes. These findings highlight the limitations of BMI-based classification alone and suggest that additional anthropometric and metabolic markers may help identify individuals with less favorable cardiometabolic characteristics.

## 1. Introduction

Obesity and cardiometabolic disorders remain leading contributors to global morbidity and mortality, with an increasing burden observed not only in older populations but also among young adults. Cardiometabolic diseases, including cardiovascular disease and type 2 diabetes, account for a substantial proportion of premature mortality worldwide and are closely linked to adiposity-related metabolic dysfunction [1,2]. Traditionally, body mass index (BMI) has been used as the primary tool for obesity classification in both clinical practice and epidemiological settings. However, growing evidence indicates that BMI provides an incomplete assessment of adiposity and may fail to capture individual variability in body composition and metabolic risk [3,4].

BMI does not differentiate between fat mass and lean mass, nor does it account for the distribution of adipose tissue, particularly visceral fat, which is more strongly associated with insulin resistance, systemic inflammation, and cardiovascular risk. Several studies have demonstrated that reliance on BMI alone may lead to misclassification of individuals with clinically relevant adiposity and cardiometabolic alterations, especially in populations with normal BMI but unfavorable fat distribution [5,6]. In this context, alternative markers such as body fat percentage, waist-to-height ratio, and measures of visceral adiposity have been proposed as more informative indicators of metabolic risk [7,8,9].

The concept of normal-weight obesity (NWO) has emerged to describe individuals with a normal BMI but excess body fat or central adiposity. This phenotype is increasingly recognized as clinically relevant, as it is associated with a higher prevalence of cardiometabolic abnormalities despite the absence of overt obesity by BMI criteria [10,11,12,13,14]. Individuals with NWO have been shown to exhibit adverse metabolic profiles, including dyslipidemia, insulin resistance, and systemic inflammation, comparable to those observed in individuals with overt obesity [15].

The NWO phenotype is conceptually complementary to metabolically healthy obesity (MHO), a condition characterized by excess body weight but a relatively favorable metabolic profile. While MHO highlights the limitations of BMI by identifying individuals with obesity who do not exhibit the expected metabolic abnormalities, NWO represents the opposite scenario, namely individuals with normal BMI who nevertheless display increased adiposity and adverse metabolic characteristics. Together, these phenotypes illustrate the limitations of BMI-based classification and support the need for a more comprehensive assessment of body composition and metabolic health.

At the population level, the magnitude of this phenomenon is substantial. Large epidemiological studies have demonstrated that a significant proportion of individuals with normal BMI present central obesity, which is associated with increased risk of cardiovascular disease and all-cause mortality [16,17]. These findings highlight that a considerable proportion of individuals classified as low-risk based on BMI may, in fact, harbor an elevated cardiometabolic burden. Furthermore, recent evidence suggests that NWO is associated with metabolic disturbances and less favorable cardiometabolic profiles, reinforcing its potential clinical relevance [18,19].

From a pathophysiological perspective, these alterations are largely driven by excess adiposity, particularly visceral fat accumulation. Adipose tissue is now recognized as an active endocrine organ capable of modulating metabolic, inflammatory, and hormonal pathways. Excess fat mass, even in individuals with normal BMI, can promote insulin resistance, dyslipidemia, and chronic low-grade inflammation, thereby contributing to the early stages of cardiometabolic disease [20]. These processes often remain clinically silent for years, especially in young adults, delaying diagnosis and preventive interventions.

Young populations represent a particularly relevant group in this context. Cardiometabolic risk factors frequently emerge during early adulthood and may remain undetected due to the limitations of conventional screening tools and the predominant reliance on BMI-based classification. Longitudinal studies have shown that metabolic abnormalities established early in life can persist into adulthood and are associated with less favorable cardiometabolic trajectories over time [21,22]. However, the identification of high-risk individuals in this age group remains challenging.

Young adults represent a particularly important population in which to investigate normal-weight obesity and its associated cardiometabolic characteristics. Although this age group is generally considered to be at relatively low cardiometabolic risk, early adiposity-related metabolic alterations may already be present and remain undetected when body mass index is used as the sole screening tool. Identifying these hidden risk profiles may contribute to improved preventive strategies and a better understanding of early cardiometabolic risk trajectories. However, large-scale studies evaluating normal-weight obesity and its cardiometabolic implications in young adults remain relatively limited.

In this context, there is a growing need to move beyond BMI-based approaches and to incorporate more comprehensive assessments of adiposity and metabolic risk. Identifying phenotypes such as normal-weight obesity may facilitate the identification of individuals with less favorable cardiometabolic profiles, particularly in young and apparently healthy populations.

Therefore, the present study aims to evaluate the prevalence of normal-weight obesity and its association with cardiometabolic risk markers in a cohort of young adults undergoing occupational health assessments, exploring the clinical implications of adiposity beyond BMI-based classification.

## 2. Methods

### 2.1. Study Design and Population

A cross-sectional observational study was conducted using data derived from routine occupational health assessments performed in a large cohort of young adults in Spain. The study population consisted of young adults undergoing routine occupational health assessments.

Participants aged between 22 and 30 years were eligible for inclusion. A total of 13,282 individuals underwent occupational health assessments during the study period. Of these, 196 were excluded because of missing key anthropometric measurements and 212 because of missing biochemical data. The final analytical cohort therefore consisted of 12,874 participants.

Overall, missing data accounted for 408 participants (3.1% of the initially eligible population). Missing values were primarily related to incomplete anthropometric or laboratory assessments. Because the proportion of missing data was low, complete-case analysis was considered unlikely to introduce substantial selection bias.

The study was designed and reported in accordance with the Strengthening the Reporting of Observational Studies in Epidemiology (STROBE) guidelines for observational research [23].

**Figure 1:**
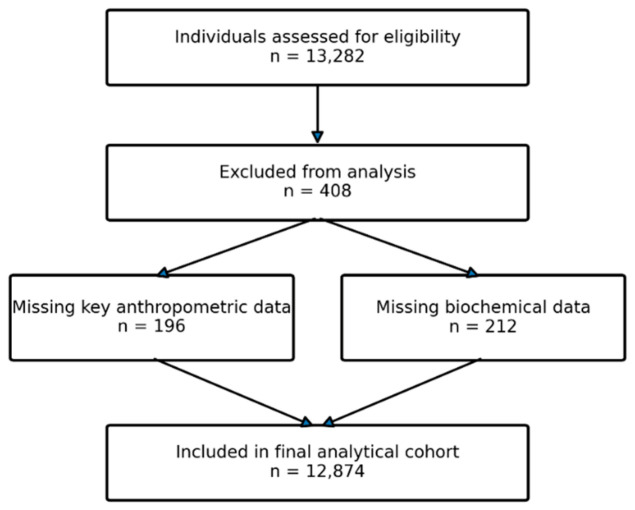
STROBE flowchart of participant selection, exclusions, and final study population.

The flow of participants through the study, including eligibility assessment, exclusions, and the final analytical sample, is presented in Figure 1.

### 2.2. Data Collection

Data were collected during standardized occupational health examinations following a structured protocol. Information on sociodemographic characteristics (age, sex, and professional group) and lifestyle factors (smoking status, physical activity, and adherence to the Mediterranean diet) was obtained through validated questionnaires.

Physical activity was assessed using standardized instruments based on international recommendations, while dietary patterns were evaluated using the 14-item Mediterranean Diet Adherence Screener (MEDAS), originally developed and validated within the PREDIMED study. These approaches have been widely used in epidemiological studies and provide reliable estimates of lifestyle-related exposures [24,25,26].

### 2.3. Anthropometric and Clinical Assessment

Anthropometric measurements were obtained by trained healthcare professionals following standardized procedures. Body weight and height were measured with participants wearing light clothing and no shoes, and body mass index (BMI) was calculated as weight in kilograms divided by height in meters squared (kg/m^2^).

Waist circumference was measured at the midpoint between the lower rib margin and the iliac crest. The waist-to-height ratio (WtHR) was calculated as waist circumference divided by height, and a cut-off value of ≥0.5 was considered indicative of central adiposity, in line with previous evidence [27].

Body composition parameters, including total body fat percentage and visceral fat estimation, were obtained using a Tanita BF-350 bioelectrical impedance analyzer (Tanita Corporation, Tokyo, Japan). Measurements were performed by trained personnel following the manufacturer’s recommendations. Participants were assessed in the morning, in a fasting state, after bladder emptying, and without intense physical activity during the previous 12 h. Standardized measurement conditions were applied to minimize hydration-related variability. Body fat percentage and visceral fat estimates were derived automatically using the proprietary prediction equations incorporated into the device software. Because of the large-scale occupational setting, the menstrual cycle phase was not systematically recorded or controlled in female participants. Bioelectrical impedance analysis is widely used in epidemiological studies due to its feasibility and acceptable validity [28].

Blood pressure was measured using validated automatic devices after a period of rest, following current clinical recommendations.

### 2.4. Definition of Phenotypes

Participants were categorized according to BMI and adiposity measures.

Normal BMI was defined as 18.5–24.9 kg/m^2^. Within this group, individuals were further classified into the following:

- Normal-weight obesity (NWO) was defined using two complementary approaches. First, a strict definition was applied, based on normal BMI (18.5–24.9 kg/m^2^) combined with central adiposity, defined as a waist-to-height ratio (WtHR) ≥ 0.5. Second, an expanded definition was used to capture a broader phenotype of hidden adiposity, defined as normal BMI together with at least one altered adiposity marker, including WtHR ≥ 0.5, increased body fat percentage (>25% in men and >35% in women), or elevated visceral fat according to the manufacturer’s recommendations. While sex-specific cut-offs were applied for body fat percentage, visceral fat classification was based on the manufacturer’s standardized scale and was not sex-specific. Because WtHR ≥ 0.5 is the defining criterion of the strict NWO definition, all individuals classified as strict NWO were necessarily included within the expanded NWO category.

We acknowledge that this strict definition based on normal BMI combined with WtHR ≥ 0.5 is conceptually closer to normal-weight central obesity than to the classical definition of normal-weight obesity based exclusively on excess body fat percentage. However, WtHR was selected because of its recognized ability to identify central adiposity and cardiometabolic risk in apparently normal-weight individuals. The expanded definition was therefore included as a complementary approach to capture a broader spectrum of hidden adiposity.

Metabolically healthy normal-weight (MHNW) individuals were defined as those with normal BMI and no evidence of excess adiposity.

Consequently, the reference MHNW group differed slightly between the strict and expanded analyses. Under the expanded definition, participants with normal BMI and WtHR < 0.5 but elevated body fat percentage or visceral fat were reclassified from MHNW to expanded NWO.

The strict definition was used for the primary analysis, while the expanded definition was applied in sensitivity analyses. Participants with BMI ≥ 25 kg/m^2^ were classified as overweight or obese according to standard criteria.

This combined approach allows identification of individuals with discordant anthropometric and adiposity profiles and has been proposed as a more accurate method for detecting hidden cardiometabolic risk [29].

### 2.5. Biochemical and Cardiometabolic Assessment

Fasting blood samples were obtained following standard laboratory procedures. The following parameters were analyzed:

- Total cholesterol;
- LDL cholesterol;
- HDL cholesterol;
- Triglycerides;
- Fasting glucose.

Derived cardiometabolic indices were calculated, including the triglyceride-to-HDL cholesterol ratio and other atherogenic indices, which have been shown to be robust markers of cardiometabolic risk and insulin resistance [30].

For logistic regression analyses, cardiometabolic outcomes were defined as follows: elevated triglycerides (≥150 mg/dL), low HDL cholesterol (<40 mg/dL in men and <50 mg/dL in women), elevated TG/HDL ratio (>2), and impaired fasting glucose (≥100 mg/dL). These thresholds were selected according to commonly used clinical and epidemiological criteria for cardiometabolic risk assessment.

### 2.6. Statistical Analysis

Continuous variables were assessed for normality using graphical inspection and distributional diagnostics. Because the main study variables showed approximately normal distributions in this large sample, results are presented as mean ± standard deviation, and comparisons were performed using Student’s *t*-test or analysis of variance (ANOVA), as appropriate. Categorical variables were compared using chi-squared tests.

Multivariable logistic regression models were constructed to assess the association between normal-weight obesity and adverse cardiometabolic profiles. Models were adjusted for age and sex.

Age and sex were selected a priori as potential confounding variables because of their well-established associations with adiposity distribution and cardiometabolic risk. Additional lifestyle variables, including smoking status, physical activity, and Mediterranean diet adherence, were collected for descriptive purposes but were not included in the primary regression models. Likewise, the professional group was described at baseline but was not included as an adjustment variable in the multivariable models. Because only age and sex were entered simultaneously into the models, collinearity was not considered a relevant concern. BMI, WtHR, and lipid-related variables were not simultaneously included as independent covariates in the regression models; therefore, assessment of multicollinearity among these variables was not required.

Sex-stratified analyses were performed to explore whether the associations between normal-weight obesity and cardiometabolic risk markers differed between men and women. Formal interaction tests were not conducted. Statistical significance was set at a two-sided *p*-value < 0.05. All statistical analyses were performed using IBM SPSS Statistics for Windows, version 30.0 (IBM Corp., Armonk, NY, USA). Data were systematically checked for completeness and consistency prior to analysis. Continuous variables were assessed for normality, and appropriate parametric or non-parametric tests were applied accordingly. The use of SPSS ensured standardized data handling and robust statistical processing throughout the study.

All analyses were performed using both definitions of NWO. Primary analyses were based on the strict definition, while sensitivity analyses using the expanded definition were conducted.

For graphical representation of continuous variables, boxplots were constructed using Tukey’s method, with whiskers extending to 1.5 times the interquartile range (IQR). Observations beyond this range were displayed as outliers but were retained in the analyses because they represented plausible biological values, and no data-entry errors were identified.

A post hoc power analysis was performed for the main cardiometabolic outcomes comparing strict NWO and MHNW participants, using a two-sided alpha level of 0.05. Despite the relatively small number of strict NWO participants, statistical power was adequate for the main outcomes, particularly elevated triglycerides, elevated TG/HDL ratio, and impaired fasting glucose. Power was lower for low HDL cholesterol in the overall analysis, and therefore, this specific result should be interpreted with caution.

A post hoc power analysis was performed for the principal cardiometabolic outcomes. The results are presented in Supplementary Table S1.

## 3. Results

### 3.1. Population Characteristics

A total of 12,874 participants were included in the final analysis. The mean age of the study population was 26.0 years, reflecting a homogeneous cohort of young adults. Women represented the majority of the sample (68.4%), while men accounted for 31.6%.

Most participants fell within the normal BMI range, with 9290 individuals (72.2%) classified as having normal weight. This confirms that the study population was predominantly composed of individuals traditionally considered at low cardiometabolic risk based on BMI.

The baseline characteristics of the study population are summarized in Table 1.

### 3.2. Prevalence of Normal-Weight Obesity

Among individuals with normal BMI, the prevalence of normal-weight obesity (NWO) varied depending on the definition applied.

Using the strict definition based on waist-to-height ratio (WtHR ≥ 0.5), the prevalence was 1.79% (*n* = 166).

When applying the expanded definition, which incorporated additional adiposity markers, the prevalence increased to 2.30% (*n* = 214), identifying a larger subgroup of individuals with hidden adiposity despite normal BMI.

Differences in anthropometric and cardiometabolic parameters between metabolically healthy normal-weight individuals and those with strict normal-weight obesity are presented in Table 2.

Participants with strict NWO exhibited a less favorable anthropometric and cardiometabolic profile than MHNW individuals, including higher measures of central adiposity and adverse lipid and glucose parameters (Table 2). No significant difference was observed for visceral fat.

Although this prevalence appears modest, it is clinically relevant given the young age of the population and the expected low baseline risk. Importantly, this subgroup represents individuals who would be systematically misclassified as low-risk using BMI alone.

### 3.3. Derived Cardiometabolic Indices

**Figure 2:**
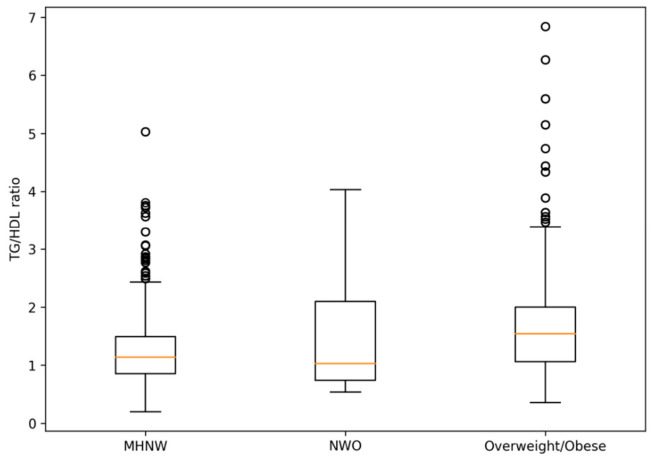
Distribution of the triglyceride-to-HDL ratio according to adiposity-defined phenotypes. Boxplots represent the median and interquartile range of the TG/HDL ratio in metabolically healthy normal-weight (MHNW), normal-weight obesity (NWO), and overweight/obese individuals. Boxplots represent the median, interquartile range (IQR), and whiskers extending to 1.5× IQR according to Tukey’s method. Points beyond the whiskers are displayed individually as outliers.

The distribution of TG/HDL ratio values across adiposity-defined phenotypes is shown in Figure 2.

As shown in Figure 2, individuals with normal-weight obesity exhibited higher TG/HDL ratio values than metabolically healthy normal-weight participants, approaching those observed in overweight and obese individuals, thereby reinforcing the presence of an unfavorable cardiometabolic profile despite a normal BMI.

### 3.4. Multivariable Analysis

The association between strict normal-weight obesity and adverse cardiometabolic profiles was further evaluated using multivariable logistic regression models (Table 3).

Multivariable logistic regression analyses confirmed significant associations between strict NWO and several adverse cardiometabolic outcomes, particularly elevated triglycerides, TG/HDL ratio, and impaired fasting glucose (Table 3).

**Figure 3:**
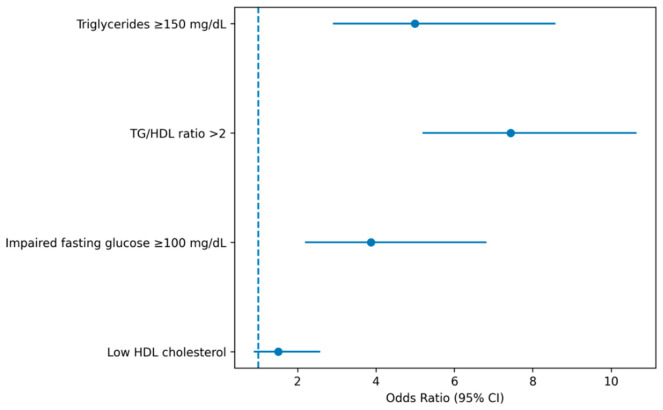
Association between strict normal-weight obesity and cardiometabolic risk markers (multivariable logistic regression analysis). Odds ratios (ORs) and 95% confidence intervals (CIs) are shown. Models were adjusted for age and sex.

Figure 3 illustrates the multivariable-adjusted associations between strict normal-weight obesity and cardiometabolic risk markers. After adjustment for age and sex, strict normal-weight obesity was independently associated with an increased likelihood of all cardiometabolic abnormalities examined. The highest odds were observed for a TG/HDL ratio > 2, followed by elevated triglycerides and impaired fasting glucose, whereas the association with low HDL cholesterol was weaker but remained statistically significant.

### 3.5. Sex-Specific Analysis

Sex-stratified analyses of the association between strict normal-weight obesity and cardiometabolic risk markers are presented in Table 4.

Sex-stratified analyses confirmed that the association between normal-weight obesity and adverse cardiometabolic profiles was present in both men and women. However, the magnitude of the associations was consistently higher in men, particularly for triglycerides and the TG/HDL ratio. These findings suggest that central adiposity may exert a stronger metabolic impact in men, even at young ages, although the pattern of increased risk is evident across both sexes.

### 3.6. Sensitivity Analysis

Sensitivity analyses confirmed the robustness of the results.

To further assess the robustness of the findings, anthropometric and cardiometabolic characteristics were re-evaluated using the expanded definition of normal-weight obesity (Table 5).

Using alternative adiposity definitions yielded similar trends, with NWO consistently associated with a worse cardiometabolic profile.

### 3.7. Summary of Findings

Overall, the results demonstrate that:

- A measurable proportion of young adults with normal BMI present central adiposity.
- This phenotype is associated with an unfavorable cardiometabolic profile.
- BMI alone fails to identify this subgroup.
- Adiposity-based measures provide additional clinical value.

## 4. Discussion

### 4.1. Principal Findings

This study demonstrates that a measurable proportion of young adults classified as having normal BMI exhibit a distinct phenotype characterized by increased adiposity and early cardiometabolic alterations. Individuals with normal-weight obesity (NWO) showed consistently higher values of triglycerides, glucose, and atherogenic lipid indices compared with metabolically healthy normal-weight participants, despite sharing similar BMI ranges.

Importantly, the triglyceride-to-HDL cholesterol ratio emerged as the variable most strongly associated with NWO in multivariable models, suggesting that this index may capture less favorable metabolic alterations more effectively than isolated lipid parameters. These findings were consistent across sensitivity analyses using an expanded definition of NWO and remained evident in sex-stratified analyses, supporting the robustness of the observed associations.

Although several between-group differences reached statistical significance, their absolute magnitude was modest for some variables, particularly blood pressure and triglyceride concentrations. Given the large sample size of the present study, statistical significance should not necessarily be interpreted as indicating large clinical effects. Nevertheless, the consistent direction of the associations across multiple anthropometric, lipid, and glycemic markers, together with the strong odds ratios observed for elevated triglycerides, TG/HDL ratio, and impaired fasting glucose, supports the presence of a less favorable cardiometabolic profile among individuals with NWO.

### 4.2. Comparison with Previous Research and NWO Phenotype

Our results are consistent with a growing body of evidence indicating that BMI-based classification fails to identify a substantial proportion of individuals with excess adiposity and increased cardiometabolic risk. The concept of normal-weight obesity has been increasingly recognized as a clinically relevant phenotype associated with insulin resistance, dyslipidemia, and increased cardiovascular risk [31,32].

Previous studies have demonstrated that individuals with normal BMI but elevated body fat percentage exhibit metabolic alterations comparable to those observed in overweight populations [33,34]. Our findings extend this evidence to a large cohort of young adults undergoing occupational health assessments, highlighting that these alterations are already present at early ages and may remain undetected when BMI is used as the sole screening tool.

The prevalence of NWO observed in our study (1.79–2.30%) was lower than that reported in the recent systematic review and meta-analysis by Sruthi et al., which identified substantially higher prevalence estimates across diverse international populations. Differences in age distribution, occupational characteristics, ethnicity, and NWO definitions may partly explain this discrepancy.

### 4.3. Role of Adiposity Distribution and Early Metabolic Alterations

The observed associations are likely related to differences in adiposity distribution rather than overall body weight. Central adiposity, as reflected by the waist-to-height ratio, has been consistently associated with insulin resistance, dyslipidemia, and adverse cardiometabolic profiles in previous studies [35,36]. Although visceral adiposity is also considered metabolically relevant, visceral fat estimates did not differ significantly between MHNW and strict NWO participants in the present study and therefore should be interpreted with caution.

In this context, indices such as waist-to-height ratio and body composition measures provide a more accurate representation of cardiometabolic risk than BMI alone. The consistent differences observed in TG/HDL ratio and glucose levels in NWO individuals support the hypothesis of less favorable metabolic characteristics even in the absence of overt weight excess, reinforcing the need for more sensitive screening strategies [37,38].

An interesting finding was that visceral fat estimates obtained by bioelectrical impedance analysis did not differ significantly between strict NWO and MHNW participants, whereas significant differences emerged when the expanded NWO definition was applied. This apparent discrepancy may be explained by the fact that the strict definition was based exclusively on waist-to-height ratio, whereas the expanded definition incorporated additional adiposity markers, including elevated visceral fat. Furthermore, bioelectrical impedance-derived visceral fat estimates may have limited sensitivity for detecting subtle differences in visceral adiposity among young normal-weight individuals. Therefore, the waist-to-height ratio may capture early central fat accumulation that is not fully reflected by bioelectrical impedance-derived visceral fat measurements.

### 4.4. TG/HDL Ratio as a Key Marker of Hidden Risk

One of the most relevant findings of this study is the strong association between NWO and the TG/HDL ratio, which showed the highest effect size among all evaluated biomarkers. This index has been widely proposed as a surrogate marker of insulin resistance and atherogenic dyslipidemia, with demonstrated predictive value for cardiometabolic risk in young populations [39,40].

The progressive increase in TG/HDL values observed across phenotypes—from metabolically healthy normal-weight individuals to NWO and overweight/obese groups—supports the concept of a metabolic continuum that is not captured by BMI classification alone. These findings reinforce the clinical utility of the TG/HDL ratio as a simple and accessible tool for early risk identification [41,42].

### 4.5. Sex Differences and Biological Plausibility

Sex-stratified analyses revealed that the association between NWO and adverse cardiometabolic profiles was present in both men and women. Although numerically higher odds ratios were observed in men for several outcomes, formal interaction analyses were not performed, and therefore, these differences should be interpreted cautiously. This pattern is biologically plausible and may be related to sex-specific differences in fat distribution and metabolic regulation described in previous studies [43,44]. However, the present study was not specifically designed to evaluate the mechanisms underlying these sex differences, and visceral fat estimates did not differ significantly between MHNW and strict NWO participants. Therefore, the biological explanation should be considered hypothesis-generating rather than demonstrative.

In contrast, women generally exhibit a more favorable fat distribution pattern at younger ages, with greater subcutaneous fat deposition, which may partially attenuate metabolic risk. However, the persistence of significant associations in both sexes indicates that NWO represents a relevant phenotype regardless of sex, although its clinical expression may differ.

Future studies should further explore sex-specific mechanisms underlying normal-weight obesity and evaluate whether different screening approaches may be required for men and women.

### 4.6. Clinical Implications: Beyond BMI-Based Screening

From a clinical perspective, these findings highlight the limitations of BMI as a standalone screening tool in young populations. In our cohort, all individuals with NWO would have been classified as low risk based on BMI, despite presenting clear metabolic alterations.

The incorporation of simple anthropometric indices such as waist-to-height ratio, together with derived lipid markers such as TG/HDL ratio, may substantially improve the identification of individuals with potentially unfavorable cardiometabolic profiles who would otherwise be classified as low risk according to BMI alone. In the present study, a WtHR threshold of ≥0.5 was used to define central adiposity, consistent with previous literature. However, the present analysis was not designed to establish clinical cut-off values for the TG/HDL ratio or to evaluate their incremental prognostic value beyond established cardiovascular risk algorithms. Therefore, these markers should be considered complementary tools that may support risk assessment within existing prevention frameworks, including those recommended by current ESC cardiovascular prevention guidelines.

Furthermore, the concept of normal-weight obesity underscores the need to shift from weight-centered approaches toward a more comprehensive evaluation of body composition and metabolic health.

### 4.7. Strengths and Limitations

This study has several strengths. It includes a large and well-characterized cohort of young adults, allowing the identification of early cardiometabolic alterations in a population often considered low-risk. The simultaneous evaluation of anthropometric, body composition, and biochemical parameters provides a comprehensive assessment of the NWO phenotype. Additionally, the use of both strict and expanded definitions of NWO strengthens the robustness of the findings.

Although the overall sample size was large, the number of participants classified as strict NWO was comparatively small, particularly in sex-stratified analyses. Nevertheless, post hoc power analyses demonstrated adequate statistical power for the principal cardiometabolic outcomes evaluated, supporting the robustness of the observed associations.

However, some limitations should be acknowledged. The cross-sectional design precludes causal inference, and longitudinal studies are needed to determine the long-term clinical implications of NWO in young populations. Body composition was assessed using bioelectrical impedance analysis, which, although practical for large studies, may be less precise than imaging-based methods. Finally, the study population consisted of young occupationally active adults from a Spanish occupational health setting, which may limit the generalizability of the findings to other age groups, non-working populations, or populations from different geographic and healthcare contexts.

In addition, participants were recruited through routine occupational health assessments. Therefore, the study population may not be fully representative of all young adults, particularly individuals who are unemployed, not engaged in the workforce, or not covered by occupational health surveillance programs. Consequently, caution is warranted when extrapolating these findings to the broader young adult population. Their greater health awareness, occupational characteristics, and access to preventive healthcare services may influence both lifestyle behaviors and cardiometabolic risk profiles. Furthermore, recruitment during routine occupational health examinations may have introduced a degree of selection bias, as individuals outside the workforce or without access to occupational health surveillance were not represented. Therefore, caution is warranted when extrapolating these findings to the broader young adult population.

In addition, the multivariable regression models were adjusted only for age and sex. Although information on smoking status, physical activity, and adherence to the Mediterranean diet was collected, these variables were not included in the primary models. Therefore, residual confounding by lifestyle-related factors cannot be excluded and should be considered when interpreting the observed associations.

Although the strict NWO group was relatively small (n = 166), post hoc power analyses demonstrated adequate statistical power for the principal outcomes evaluated (Supplementary Table S1).

## 5. Conclusions

Normal-weight obesity represents a clinically relevant phenotype in young adults, characterized by increased adiposity and unfavorable cardiometabolic characteristics despite a normal BMI. Individuals with NWO consistently exhibited an unfavorable metabolic profile, particularly reflected by alterations in triglycerides, glucose, and atherogenic lipid indices.

The triglyceride-to-HDL cholesterol ratio emerged as a marker consistently associated with an unfavorable cardiometabolic profile and showed the strongest association with NWO among the metabolic markers evaluated in the present study. These findings suggest the presence of metabolic alterations that may not be captured by BMI-based classification alone.

Importantly, the results were consistent across different definitions of NWO and in sex-stratified analyses, supporting the robustness of the observed associations and reinforcing the clinical relevance of this phenotype in both men and women.

From a clinical perspective, these findings underscore the limitations of BMI as a standalone screening tool and support the consideration of additional anthropometric and metabolic markers, such as waist-to-height ratio and TG/HDL ratio, as complementary tools for cardiometabolic assessment. However, the present cross-sectional study was not designed to determine their incremental prognostic value beyond established risk assessment strategies. Early identification of individuals with hidden adiposity may help identify young adults presenting a less favorable cardiometabolic profile despite a normal BMI.

In conclusion, moving beyond BMI-based approaches toward a more comprehensive evaluation of adiposity and metabolic health may improve the identification of unfavorable cardiometabolic characteristics in apparently healthy individuals. However, prospective studies are needed to determine whether these findings translate into improved risk prediction and preventive strategies.

## Figures and Tables

**Table 1:** Baseline characteristics of the study population.

Variable	Total (*n* = 12,874)
Age (years)	26.0 ± 2.0
**Sex**	
Female, *n* (%)	8810 (68.4)
Male, *n* (%)	4064 (31.6)
**Anthropometric parameters**	
BMI (kg/m^2^)	22.7 ± 3.7
Waist circumference (cm)	73.9 ± 9.2
Waist-to-height ratio	0.44 ± 0.05
**Body composition**	
Body fat (%)	20.1 ± 7.5
Visceral fat	1.9 ± 1.6
**Lifestyle factors**	
Current smokers, *n* (%)	1939 (15.1)
Physically active, *n* (%)	7332 (56.9)
Mediterranean diet adherence, *n* (%)	6797 (52.8)

**Table 2:** Anthropometric and cardiometabolic characteristics according to strict normal-weight obesity status.

Variable	MHNW (*n* = 9124)	Strict NWO (*n* = 166)	*p*-Value
Age (years)	26.0 ± 1.9	26.3 ± 1.8	0.006
Female, *n* (%)	6526 (71.5)	104 (62.7)	0.016
Male, *n* (%)	2598 (28.5)	62 (37.3)	0.016
BMI (kg/m^2^)	21.6 ± 1.8	22.2 ± 1.3	<0.001
Waist circumference (cm)	71.3 ± 6.6	84.2 ± 7.0	<0.001
Waist-to-height ratio	0.43 ± 0.03	0.52 ± 0.04	<0.001
Systolic BP (mmHg)	113.9 ± 9.2	117.0 ± 10.8	<0.001
Diastolic BP (mmHg)	69.1 ± 7.1	70.5 ± 8.1	0.032
Total cholesterol (mg/dL)	173.7 ± 26.3	192.9 ± 27.9	<0.001
LDL cholesterol (mg/dL)	117.0 ± 22.4	125.6 ± 30.1	<0.001
HDL cholesterol (mg/dL)	59.5 ± 10.8	54.0 ± 6.6	<0.001
Triglycerides (mg/dL)	69.6 ± 27.8	77.7 ± 39.5	0.010
Glucose (mg/dL)	83.6 ± 10.1	90.7 ± 8.6	<0.001
TG/HDL ratio	1.21 ± 0.54	1.54 ± 1.03	<0.001
Total cholesterol/HDL ratio	3.00 ± 0.64	3.69 ± 1.11	<0.001
LDL/HDL ratio	1.75 ± 0.59	2.38 ± 0.94	<0.001
Body fat (%)	19.4 ± 6.5	23.2 ± 4.0	0.003
Visceral fat	1.5 ± 0.8	1.6 ± 0.5	0.699

Data are expressed as mean ± standard deviation or number (percentage). MHNW: metabolically healthy normal-weight; NWO: normal-weight obesity; BMI: body mass index; BP: blood pressure; TG: triglycerides; HDL: high-density lipoprotein; LDL: low-density lipoprotein.

**Table 3:** Multivariable logistic regression analysis of the association between strict normal-weight obesity and cardiometabolic risk markers.

Outcome	OR (95% CI)	*p*-Value
Elevated triglycerides (≥150 mg/dL)	4.99 (2.90–8.58)	<0.001
Low HDL cholesterol	1.51 (0.88–2.58)	0.132
Elevated TG/HDL ratio (>2)	7.44 (5.19–10.65)	<0.001
Impaired fasting glucose (≥100 mg/dL)	3.87 (2.19–6.82)	<0.001

Models were adjusted for age and sex. Low HDL cholesterol was defined as <40 mg/dL in men and <50 mg/dL in women.

**Table 4:** Sex-stratified analysis of the association between strict normal-weight obesity and cardiometabolic risk markers.

**Outcome**	**Men OR (95% CI)**	***p*** **-Value**	**Women OR (95% CI)**	***p*** **-Value**
Elevated triglycerides	5.82 (2.90–11.67)	<0.001	4.31 (2.12–8.75)	<0.001
Low HDL cholesterol	1.72 (0.89–3.32)	0.106	1.39 (0.73–2.65)	0.314
Elevated TG/HDL ratio	8.21 (5.10–13.21)	<0.001	6.87 (4.21–11.20)	<0.001
Impaired fasting glucose	4.65 (2.20–9.81)	<0.001	3.12 (1.58–6.15)	0.001

Models were adjusted for age. Low HDL cholesterol was defined as <40 mg/dL in men and <50 mg/dL in women.

**Table 5:** Anthropometric and cardiometabolic characteristics according to the expanded normal-weight obesity definition (sensitivity analysis).

Variable	MHNW (*n* = 9076)	Expanded NWO (*n* = 214)	*p*-Value
Age (years)	26.0 ± 1.9	25.8 ± 2.0	0.222
Female, *n* (%)	6522 (71.9)	108 (50.5)	<0.001
Male, *n* (%)	2554 (28.1)	106 (49.5)	<0.001
BMI (kg/m^2^)	21.6 ± 1.8	22.8 ± 1.3	<0.001
Waist circumference (cm)	71.3 ± 6.6	81.9 ± 7.1	<0.001
Waist-to-height ratio	0.43 ± 0.03	0.49 ± 0.05	<0.001
Body fat (%)	19.3 ± 6.4	27.4 ± 4.3	<0.001
Visceral fat	1.5 ± 0.8	2.2 ± 0.5	<0.001
Systolic BP (mmHg)	113.9 ± 9.2	115.8 ± 9.6	0.005
Diastolic BP (mmHg)	69.2 ± 7.1	69.6 ± 8.4	0.449
Total cholesterol (mg/dL)	173.8 ± 26.3	186.9 ± 26.8	<0.001
LDL cholesterol (mg/dL)	117.0 ± 22.4	126.8 ± 26.0	<0.001
HDL cholesterol (mg/dL)	59.5 ± 10.9	54.2 ± 6.5	<0.001
Triglycerides (mg/dL)	69.7 ± 27.8	73.3 ± 35.8	0.149
Glucose (mg/dL)	83.6 ± 10.1	88.8 ± 7.3	<0.001
TG/HDL ratio	1.22 ± 0.54	1.44 ± 0.93	<0.001
Total cholesterol/HDL ratio	3.00 ± 0.65	3.55 ± 1.02	<0.001
LDL/HDL ratio	1.75 ± 0.59	2.42 ± 0.83	<0.001

Data are expressed as mean ± standard deviation or number (percentage). Expanded normal-weight obesity was defined as normal BMI together with central adiposity (WtHR ≥ 0.5), increased body fat percentage, or elevated visceral fat. MHNW: metabolically healthy normal-weight; NWO: normal-weight obesity; BMI: body mass index; BP: blood pressure; TG: triglycerides; HDL: high-density lipoprotein; LDL: low-density lipoprotein.

## Data Availability

This manuscript was prepared in accordance with the Strengthening the Reporting of Observational Studies in Epidemiology (STROBE) guidelines for cross-sectional studies. The data supporting the findings of this study are included within the article. Additional information is available from the corresponding author upon reasonable request.

## Supplementary Materials

The following supporting information can be downloaded at: https://www.mdpi.com/article/10.3390/medsci14030354/s1, Table S1: Post-hoc statistical power analysis for the principal outcomes.
