# Supplementary material for: Normal-Weight Obesity and Hidden Cardiometabolic Risk in Young Adults: Implications Beyond BMI-Based Classification
Source: Med Sci (Basel). 2026 Jun 27;14(3):354. doi: 10.3390/medsci14030354 (PMC13414445; doi:10.3390/medsci14030354)
Supplement: Supplementary file 1 [file medsci-14-00354-s001.zip › medsci-4328394-supplementary.pdf]

Supplementary Materials: Normal-Weight Obesity and Hidden Cardiometabolic Risk in Young Adults: Implications Beyond BMI-Based Classification

Alberto Ramírez Gallegos, Pedro Juan Tárraga López, Mónica Silu Piña Dabreu, Lluis Rodas Cañellas, Ángel Arturo López-González and José Ignacio Ramírez-Manent

Table S1. Post-hoc statistical power analysis for the principal outcomes.

Outcome	Overall power (%)	Men (%)	Women (%)
Elevated triglycerides	98.8	100.0	79.9
Low HDL cholesterol	6.5	100.0	100.0
Elevated TG/HDL ratio	100.0	100.0	85.5
Impaired fasting glucose	100.0	96.9	100.0
